# Parasitic nematodes of the genus *Syphacia* Seurat, 1916 infecting Cricetidae in the British Isles: the enigmatic status of *Syphacia nigeriana*

**DOI:** 10.1017/S0031182021001578

**Published:** 2022-01

**Authors:** Jerzy M. Behnke, Alex Stewart, Lesley Smales, Gemma Cooper, Ann Lowe, John M. Kinsella, Anna Bajer, Dorota Dwużnik-Szarek, Jeremy Herman, Jonathan Fenn, Stefano Catalano, Christophe A. Diagne, Joanne P. Webster

**Affiliations:** 1 School of Life Sciences, University of Nottingham, University Park, Nottingham NG7 2RD, UK; 2 Faculty of Health and Medical Sciences, University of Surrey, Guildford GU2 7XH, UK; 3 South Australian Museum, North Terrace, Adelaide, SA 5000, Australia; 4 HelmWest laboratory, 2108 Hilda Avenue, Missoula, MT, 59801, USA; 5 Department of Eco-Epidemiology of Parasitic Diseases, Faculty of Biology, Institute of Developmental Biology and Biomedical Sciences, University of Warsaw, 1 Miecznikowa Street, 02-096, Warsaw, Poland; 6 Department of Natural Sciences, National Museums Scotland, Chambers Street, Edinburgh EH1 1JF, UK; 7 Moredun Research Institute, Pentland Science Park, Bush Loan, Penicuik EH26 0PZ, UK; 8 Department of Pathobiology and Population Sciences, Royal Veterinary College, University of London, Hawkshead Campus, Herts, AL9 7TA, UK; 9 CBGP, IRD, CIRAD, INRAE, Montpellier SupAgro, University of Montpellier, 755 Avenue du Campus Agropolis, 34988 Montferrier-sur-Lez, Cedex, France

**Keywords:** *Clethrionomys glareolus*, *cox-1* gene, *Microtus agrestis*, *Myodes glareolus*, oxyurid nematodes, rDNA (ITS-1-5.8S-ITS2), 18S rDNA, *Syphacia nigeriana*, *Syphacia petrusewiczi*

## Abstract

Oxyurid nematodes (*Syphacia* spp.) from bank (*Myodes glareolus)* and field/common (*Microtus* spp.) voles, from disparate geographical sites in the British Isles, were examined morphologically and genetically. The genetic signatures of 118 new isolates are provided, based primarily on the rDNA internal transcribed spacers (ITS1-5.8S-ITS2) region and for representative isolates also on the small subunit 18S rDNA region and cytochrome *c* oxidase subunit 1 (*cox-1*) gene locus. Genetic data on worms recovered from *Microtus* spp. from the European mainland and from other rodent genera from the Palaearctic, North America and West Africa are also included. We test historical hypotheses indicating that *S. nigeriana* is a generalist species, infecting a range of different rodent genera. Our results establish that *S. nigeriana* is a parasite of both bank and field voles in the British Isles. An identical genotype was also recorded from Hubert's multimammate mouse (*Mastomys huberti*) from Senegal, but *Mastomys* spp. from West Africa were additionally parasitized by a related, although genetically distinct *Syphacia* species. We found no evidence for *S. petrusewiczi* in voles from the British Isles but isolates from Russia and North America were genetically distinct and formed their own separate deep branch in maximum likelihood molecular phylogenetic trees.

## Graphical abstract

**Figure:**
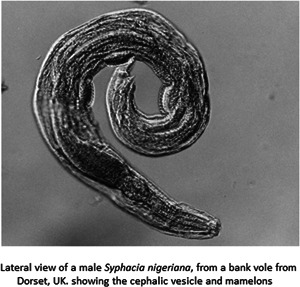

## Introduction

Nematodes of the genus *Syphacia* Seurat, 1916 (Oxyuridae Cobbold, 1864: Syphaciinae Railliet, 1916) are among the most common members of the helminth communities in wild rodents worldwide (Roman, 1951), but perhaps best known are the two species that parasitize laboratory rodents and their free-living conspecifics: *S. obvelata* (Rudolphi, 1802) in house mice (*Mus* spp.) and *S. muris* Yamaguti, 1935 in rats (*Rattus* spp.). In the western Palearctic wood mice (also referred to as long-tailed field mice, *Apodemus sylvaticus*) and yellow-necked field mice (*A. flavicollis*) are parasitized by two species, *S. stroma* (Linstow, 1884) Morgan, 1932 and *S. frederici* Roman, 1945. Sympatric voles are also typically parasitized by three species, *S. petrusewiczi* Bernard, 1966 in bank voles (*Myodes glareolus*), *S. montana* Yamaguti, 1943 in European snow voles (*Chionomys nivalis*) and European pine voles (*Microtus subterraneus*; see Tenora *et al*., 1974) and *S. nigeriana* Baylis, 1928 in other *Microtus* spp. [*M. agrestis* (northern short-tailed field vole), *M. rozianus* (Portuguese field vole), *M. lavernedii* (Mediterranean field vole), *M. arvalis* (common vole) and *M. oeconomus* (root or tundra vole)]. In addition to the seven listed here, four other species of *Syphacia* have also been recorded from European rodents, namely *S. arvicolae* Sharpilo, 1973, *S. vandenbrueli* Bernard, 1966, *S. agraria*, Sharpilo, 1973 and the fourth *S. baylisi*, which is a synonym of *S. muris* (see Tenora and Mészáros, 1975). The hosts of *S. vandenbrueli* and *S. arvicolae* (the Eurasian harvest mouse, *Micromys minutus* and the European water vole, *Arvicola* spp., respectively), are endemic in the British Isles but to the best of our knowledge their *Syphacia* spp. have never been recorded locally, although it is worth noting that Tenora *et al*. (1979*a*) recommended that this latter species should be synonymized with *S. nigeriana*. The striped field mouse, *Apodemus agrarius* (host of *S. agraria*) does not exist in the wild in the British Isles.

*Syphacia nigeriana* was first described by Baylis (1928) on the basis of worms recovered from five species of West African rodents *Taterillus gracilis* (slender gerbil from the district of Kano, Nigeria), *Gerbilliscus kempi* (northern savanna gerbil from Ibadan, Nigeria as *Taterona kempi*), *Praomys tulbergi* (Tullberg's soft-furred mouse from Adu, Nigeria), *Mastomys erythroleucus* (Guinea multimammate mouse from Ife, Nigeria) and *Lemniscomys striatus* (typical striped grass mouse from Adu, Nigeria). Although he did not designate types in his paper, specimens of *Syphacia* in Baylis' material labelled as from *T. kempi* from Ibadan, Nigeria registered in the Natural History Museum London (NHML) as 129.1.24. 26, 27, 31 are labelled paratypes (specimens additional to the holotype on which the description is based).

Subsequently, after studying specimens from a wide range of hosts, and extending the host record list to *Microtus* spp. from North America and adding six additional rodent hosts from Africa, Quentin (1971) wrote: ‘As a consequence we think that the *Syphacia* of holarctic *Microtus*, which present the cephalic and genital structures morphologically identical to those from *Syphacia* of Gerbillidae and African Muridae, belong to the same species, *Syphacia nigeriana*’ (Translation from the original in French, Quentin, 1971, p. 32).

Thereafter, European parasitologists considered the *Syphacia* species parasitizing European rodents of the genus *Microtus* to be mainly *S. nigeriana* (see Sharpilo, 1973; Tenora and Mészáros, 1975; Mészáros, 1977; Tenora *et al*., 1978; Mészáros and Murai, 1979; Tenora *et al*., 1991; Tenora and Staněk, 1994, 1995). Accordingly, *S. nigeriana* has been recorded in *M. agrestis* in Denmark (Tenora *et al*., 1991), Norway (Wiger *et al*., 1978; Tenora *et al*., 1979*b*), Finland (Tenora *et al*., 1983; Haukisalmi *et al*., 1994), in *M. rozianus* or *M. lavernedii* in Spain (as *M. agrestis*, Mas-Coma *et al*., 1978; Feliu *et al*., 1997), in *M. arvalis* in Romania (Mészáros and Murai, 1979), in *M. oeconomus* in Norway (Tenora *et al*., 1977), in *C. nivalis* in Spain (Mas-Coma *et al*., 1978) and as an occasional parasite of *M. glareolus* in Norway (Tenora *et al*., 1977, 1978; Tenora and Mészáros, 1975; Wiger *et al*., 1976) and Hungary (Mészáros, 1977).

Like most oxyurid nematodes, species of *Syphacia* are believed to show strong co-evolutionary relationships with their hosts, and hence are considered to be generally host-specific (Adamson, 1989; Hugot, 1999; Garcia *et al*., 2018). It was our view, given the distance and the intervening terrain involved, that it was highly unlikely that the parasite in European *Microtus* spp. was panmictic with those parasitizing wild rodents of quite different host genera in Africa. On these grounds, we questioned whether the species of *Syphacia* found in European *Microtus* spp. had been erroneously assigned as *S. nigeriana* and considered that a genetic analysis was warranted.

*Syphacia petrusewiczi* was described by Bernard (1966) from bank voles, *M. glareolus*, trapped near Mikołajki in the Mazury Lake District region of north-eastern Poland, where long-term surveys of bank vole helminth communities have been conducted in more recent years (Grzybek *et al*., 2015). Specimens of *S. petrusewiczi* in bank voles from this region of Poland therefore may be regarded as having come from close to the type locality (Bernard, 1966). *Syphacia petrusewiczi* is regarded as a *Myodes* specialist (Tenora and Mészáros, 1975), as emphasized, for example, by Mészáros (1978). Reported locations of *S. petrusewiczi* occurring in bank voles include Finland (Haukisalmi and Henttonen, 1993), Lithuania (Mažeika *et al*., 2003; Skyrienė *et al*., 2011), northern France (Ribas Salvador *et al*., 2011), southern Italy (Milazzo *et al*., 2003), Serbia (Bjelić-Čabrilo *et al*., 2009, 2011), Spain (Feliu *et al*., 1997; Ribas *et al*., 2009) and both European and Asian Russia (Gorelysheva *et al*., 2020). Shortly after Bernard's publication (Bernard, 1966), Quentin (1969) described a variant of this species, which he regarded as sufficiently distinct to be considered a subspecies, named *S. petrusewiczi rauschi*, a parasite of *Myodes rutilus dawsoni* from Anchorage in Alaska, USA.

Female nematodes of the genus *Syphacia* are character poor, and species identity is largely based on male worms, which, although more character rich than females, are rare in most species and in some cases still unknown (Morgan, 1932; Ogden, 1971). Male worms are thought to be short-lived, inseminating females before they reach full size and then passing out of the host (Morgan, 1932; Adamson, 1994). Species-specific diagnostic characters in female worms have been described for some species (Stewart *et al*., 2018) but most are unsuitable for quantitative studies and some are difficult to detect with certainty, especially in preserved specimens, particularly if they have been fixed in 100% ethanol. Such specimens are likely to be only partially dehydrated and/or the tissues may be inadequately preserved and therefore deformed to varying extents. Hence, the literature is likely to contain cases of misidentification of species of *Syphacia* parasitizing particular hosts, and *S. obvelata* appears to be the most commonly misidentified species in this context, possibly because as a parasite of *Mus* spp. it is so well known [e.g. Sharpe (1964) and Kisielewska and Zubczewska (1973) both recorded *S. obvelata* in *M. agrestis*; Lewis (1968) recorded *S. obvelata* in *M. glareolus*]. For these reasons, published host lists, relying primarily on morphological characters, have to be treated with some caution until all the species have been fully characterized genetically and carefully re-examined morphologically.

The helminth community of *M. agrestis* in the British Isles has been only poorly studied, and as far as we are aware, to date there is only one record of *S. nigeriana* from a population of *M. agrestis* in Kielder (Turner *et al*., 2014). Interestingly, there are no wild *Microtus* spp. in Ireland and bank voles were detected for the first time only in 1964, although they are thought to have been introduced from Germany in 1926 (White *et al*., 2013). It is pertinent, however, that recently Loxton *et al*. (2016) did not recover any *Syphacia* worms from bank voles from sites in Galway in Ireland. There are few records of the helminths of *M. glareolus* from the British Isles (Elton *et al*., 1931; Sharpe, 1964; Lewis, 1968; Canning *et al*., 1973; Loxton *et al*., 2016), and to date all those authors that have recorded *Syphacia* in bank voles from sites on the British mainland have assigned them to *S. obvelata*, some even after Bernard's (1966) paper describing *S. petrusewiczi* (Lewis, 1968; Canning *et al*., 1973). There are also several reports of *S. obvelata* from bank voles (Tenora and Zejda, 1974) and *Microtus* spp. on the European mainland, which in the light of our current understanding of this species are likely also to be misidentifications (Tenora, 1972; Tenora *et al*., 1973 and citations therein; Prokopič, 1973).

In this paper, we examine morphologically *Syphacia* species isolated from both *M. glareolus* and *Microtus* spp. voles, from several disparate sites in the British Isles, and we provide the genetic signature of each isolate that we encountered, based primarily on the rDNA internal transcribed spacers (ITS1-5.8S-ITS2) region and for representative isolates also on the 18S rDNA (SSU) region and the mitochondrial cytochrome *c* oxidase subunit 1 (*cox1*) gene locus. We also include genetic data on worms recovered from rodents of the same genera from the European mainland, and from other species from North America and West Africa to help clarify the species status of *Syphacia* in voles from the British Isles. We test Baylis’ original finding (Baylis, 1928) and Quentin's subsequent work (Quentin, 1971) indicating that *S. nigeriana* is a generalist species capable of infecting a range of different rodent genera, as widely distributed as from West Africa, Europe and even from North America (Quentin, 1971).

## Materials and methods

### Host nomenclature

We refer to *M. glareolus* rather than *Clethrionomys glareolus*, following Wilson and Reeder (2005) and Carleton *et al*. (2003), and despite the recent proposal that *Clethrionomys* has priority for the generic name of all red-backed voles (Kryštufek *et al*., 2020) to maintain parity with recent publications in the field of parasite ecology. We also refer to wood mice, rather than long-tailed field mice for *A. sylvaticus*.

### Sources of isolates

**Fig. 1.**
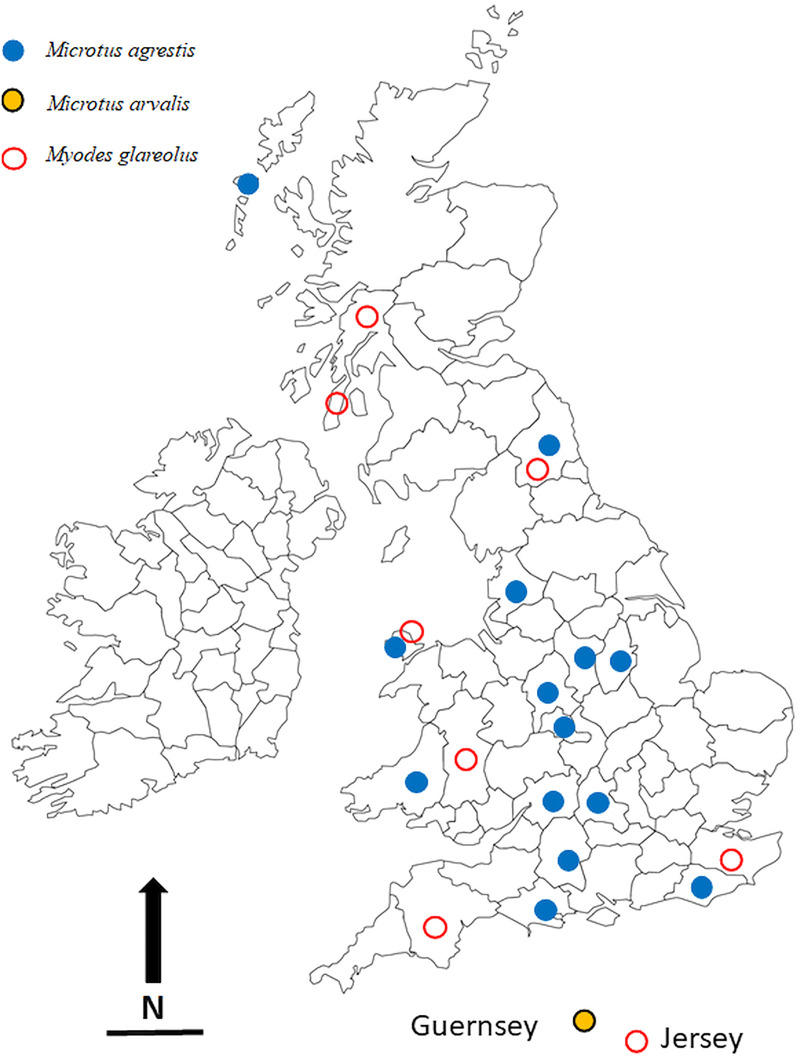
The locations in the British Isles in which rodents for this study were trapped. Symbols are plotted on the outlines of the relevant administrative counties in the British Isles in which the animals were trapped. Blue filled in circles = *Microtus agrestis*; red open circles = *Myodes glareolus*; black, yellow filled circles = *Microtus arvalis*. Scale bar = 100 km, and N = North.

The locations in which voles were trapped in the British Isles are given in Table 1 and are illustrated in Fig. 1. Most of these were obtained through field trips across the British Isles, but we also made use of a large collection of voles, both *M. agrestis* and *M. glareolus* (preserved in 100% ethanol) held at the National Museums Collection Centre, Edinburgh, UK. In addition to the voles that were infected with *Syphacia*, we also processed bank voles from 22 other locations across the country but found these to be not infected with *Syphacia*. Regardless of the source of the voles, all were examined for nematodes using the same dissection protocol and any oxyurids recovered were fixed in 100% ethanol and stored in 80–100% ethanol, and in some cases frozen, for future study. Although fixation in 100% ethanol is not ideal for morphological examination, it does allow the use of worms obtained from each host individual to be separated into two samples, one for morphological examination and one for molecular analysis. In addition, all the material registered in the Natural History Museum, London, as *S. nigeriana*, consisting of material from several hosts from African localities, was examined for comparative purposes.

**Table 1.** Isolates of *Syphacia* species genotyped in the current study

		GenBank accession numbers					MSM node		
Host	Local reference code	ITS	*cox-1*	18S	Country	Region	ITS^a^	*cox-1*^a^	18S^a^
***Apodemus* spp.**									
*A. agrarius*	POLAND-18Aag04Saf2	OK143591	–	–	Poland	Mazury	x	–	–
*A. sylvaticus*	NOTTINGHAM-12As74Sff	–	–	OK138897	England	Nottingham	–	–	b
*A. sylvaticus*	NORFOLK-12As60Ss	–	-	OK138904	England	Norfolk	–	–	d
*A. sylvaticus*	POLAND-16As-DD-24Sf	–	–	OK138899	Poland	Mazury	–	–	b
*A. sylvaticus*	WALES-GWYN-12As23Ss	–	–	OK138908	Wales	Anglesey	–	–	d
***Microtus* spp.**									
*M. agrestis*	DERBYSHIRE-1037mMagSnf	OK143582	–	–	England	Derbyshire	b	–	–
*M. agrestis*	DORSET-467mMagSnf1	OK143581	–	–	England	Dorset	a	–	–
*M. agrestis*	GLOUCESTERSHIRE-780mMagSnf2	OK143580	–	–	England	Gloucestershire	a	–	–
*M. agrestis*	LANCASHIRE-1067MSnf	OK143579	OK272535	–	England	Lancashire	a	d	–
*M. agrestis*	NORTHUMBERLAND-15MgV145Spf	OK143573	–	–	England	Northumberland	a	–	–
*M. agrestis*	NORTHUMBERLAND-15MgV145Snf	–	OK271532	–	England	Northumberland	–	d	–
*M. agrestis*	NORTHUMBERLAND-15MaV145Snf(2)	OK143577	–	–	England	Northumberland	a	–	–
*M. agrestis*	NORTHUMBERLAND-15MaV46Sn	OK143576	–	–	England	Northumberland	a	–	–
*M. agrestis*	NORTHUMBERLAND-15MaV48Snf	OK143578	OK272534	–	England	Northumberland	a	a	–
*M. agrestis*	NORTHUMBERLAND-15MaV48Spf	–	–	OK138898	England	Northumberland	–	–	a
*M. agrestis*	NORTHUMBERLAND-16MaV229Snfb	OK143572	OK272530	–	England	Northumberland	a	a	–
*M. agrestis*	NORTHUMBERLAND-15MgV108Spf	OK143574	OK272533	OK138903	England	Northumberland	a	a	a
*M. agrestis*	NORTHUMBERLAND-15MaV171Sn	OK143575	OK272531	OK138901	England	Northumberland	a	a	a
*M. agrestis*	NOTTINGHAM-19Mag02Snf	OK143552	–	–	England	Nottinghamshire	f	–	–
*M. agrestis*	NOTTINGHAM-18Mag06Snf2	OK143571	–	OK138895	England	Nottinghamshire	a	–	a
*M. agrestis*	OXFORDSHIRE-783mMagSnf2	OK143570	–	–	England	Oxfordshire	a	–	–
*M. agrestis*	STAFFORDSHIRE-894mMagSnf2	OK143566	–	–	England	Staffordshire	a	–	–
*M. agrestis*	SUSSEX-543mMagSnf2	OK143565	–	OK138890	England	Sussex	a	–	a
*M. agrestis*	W MIDLANDS-447mMagSnf2	OK143564	–	–	England	W. Midlands	a	–	–
*M. agrestis*	WILTSHIRE-995MSnf2	OK143561	OK272527	–	England	Wiltshire	a	a	–
*M. agrestis*	SCOTLAND-Uist-14ma01Sn	OK143560	OK272526	OK138902	Scotland	Hebrides, N. Uist	a	a	a
*M. agrestis*	WALES-1917mMagSnf	OK143563	–	–	Wales	Dyfed	a	–	–
*M. agrestis*	WALES-GWYN-17Mag05Snf1	OK143562	OK272528	OK138893	Wales	Anglesey	a	d	a
*M. agrestis*	POLAND-13Mag07bSn	OK143569	OK272529	–	Poland	Mazury	a	d	–
*M. agrestis*	POLAND-18Mag02Snf2	OK143568	–	–	Poland	Mazury	g	–	–
*M. agrestis*	POLAND-18MagSnf2	OK143567	–	OK138894	Poland	Mazury	h	–	a
*M. arvalis*	GUERNSEY-18Mar04Snf2	OK143558	–	–	Guernsey	Castel	c	–	–
*M. arvalis*	GUERNSEY-18Mar01Snf2	OK143559	OK272525	OK138892	Guernsey	St. Saviour	c	d	a
*M. arvalis*	POLAND-16Mar04Sn	OK143555	OK272524	–	Poland	Mazury	b	a	–
*M. arvalis*	POLAND-13Mar32Snf	OK143557	–	–	Poland	Mazury	b	–	–
*M. arvalis*	POLAND-13Mar55Sn	OK143556	–	–	Poland	Mazury	b	–	–
*M. arvalis*	POLAND-18Mar05Snf	–	–	OK138891	Poland	Mazury	–	–	a
*M. arvalis*	POLAND-18Mar05Snf2	OK143554	–	–	Poland	Mazury	h	–	*–*
*M. duodecimcostatus*	PORTUGAL-14Md02Sn	OK143553	OK272523	OK138905	Portugal	Pancas	m	f	a
***Mastomys* spp.**									
*M. huberti*	SENEGAL-Nder-SC357	OK143585	OK272538	–	Senegal	Nder	i	u	–
*M. huberti*	SENEGAL-Nder-SC349	OK143586	OK272539	–	Senegal	Nder	j	u	–
*M. huberti*	SENEGAL-RichardToll-SC32	OK143584	OK272537	–	Senegal	Richard Toll	i	u	–
*M. huberti*	SENEGAL-10MeKB6341-W03	OK143589	–	–	Senegal	Mbarigo	a	–	–
*M. huberti*	SENEGAL-10MeKB6461-W03	OK143588	OK272536	OK138896	Senegal	Mbarigo	a	g	a
*M. huberti*	SENEGAL-15MhADAL5249-W03	OK143587	OK272540	–	Senegal	Savoigne	i	u	–
*M. erythroleucus*	MALI-07MeMDD-26	OK143590	–	–	Mali	Madina-Diassa	i	–	–
*M. natalensis*	SENEGAL-CB0378 WO1	OK143583	–	–	Senegal	Kedougou	i	–	–
***Mus* spp.**									
*M. domesticus*	NOTTINGHAM-13Md01Sof	–	–	OK138907	England	Nottinghamshire	–	–	c
*M. domesticus*	STAFFORDSHIRE-18Md09So	OK143551	–	–	England	Staffordshire	r	–	–
*M. musculus*	SENEGAL-CB4044-WO	OK143548	–	–	Senegal	Dodel, Senegal valley	p	–	–
*M. musculus*	SENEGAL-CB1241-WO	OK143549	–	–	Senegal	Keur Seyni Dieng	p	–	–
*M. domesticus*	SCOTLAND-16Mm352S	–	–	OK138900	Scotland	Isle of May	–	–	c
*M. domesticus*	SCOTLAND MAY-MAYBR	OK143550	–	–	Scotland	Isle of May	q	–	–
***Myodes* spp.**									
*M. glareolus*	N DEVON-16Mg04Sp	OK143542	OK272519	OK138887	England	North Devon	a	a	a
*M. glareolus*	N DEVON-16Mg09Sp1	OK143543	OK272521	OK138885	England	North Devon	a	a	a
*M. glareolus*	N DEVON-16Mg05Sp	OK143530	–	–	England	North Devon	a	–	–
*M. glareolus*	N DEVON-16Mg05Sp(2)	OK143529	–	–	England	North Devon	a	–	–
*M. glareolus*	N DEVON-16Mg05Sp(3)	OK143528	–	–	England	North Devon	a	–	–
*M. glareolus*	KENT-05Mg979MSpf2	OK143531	–	–	England	Kent	d	–	–
*M. glareolus*	KENT-05Mg979MSpf2(2)	OK143532	–	–	England	Kent	d	–	–
*M. glareolus*	KENT-05Mg979MSpf3	OK143544	–	–	England	Kent	d	–	–
*M. glareolus*	KENT-05Mg979MSpf3(2)	OK143545	–	–	England	Kent	d	–	–
*M. glareolus*	NORTHUMBERLAND-01Mg492Spf2	OK143541	–	–	England	Northumberland	a	–	–
*M. glareolus*	JERSEY-14Mg02Sp	OK143547	OK272520	OK138888	Jersey	The Elms	b	d	a
*M. glareolus*	JERSEY-14Mg02Sp(2)	OK143546	OK272522	–	Jersey	The Elms	b	d	–
*M. glareolus*	SCOTLAND-04Mg585M1	OK143540	OK272518	OK138886	Scotland	Kintyre	a	b	a
*M. glareolus*	SCOTLAND-05Mg1156Msp	OK143539	OK272517	–	Scotland	Argyll	a	c	–
*M. glareolus*	WALES-GWYN-12Mg27Sp	OK143537	OK272516	OK138906	Wales	Anglesey	a	d	a
*M. glareolus*	WALES-GWYN-12Mg27Sp (2)	–	–	OK138889	Wales	Anglesey	–	–	a
*M. glareolus*	WALES-07Mg2017MSpf1	OK143538	–	–	Wales	Powys	e	–	–
*M. rutilus*	RUSSIA-PARA25548	OK143536	–	OK138884	Russia	Magadan Oblast	w	–	e
*M. rutilus*	USA-PARA25557	OK143535	OK272513	–	USA	Alaska	v	t	–
*M. rutilus*	USA-PARA24848	OK143533	OK272514	OK138883	USA	Yukon	u	r	–
*M. rutilus*	USA-PARA24824	OK143534	OK272515	–	USA	Yukon	u	s	f

a Letters correspond to nodes in Figs 2B, 3B and 4B. Within each column isolates with the same letter have identical sequences.

**Fig. 2.**
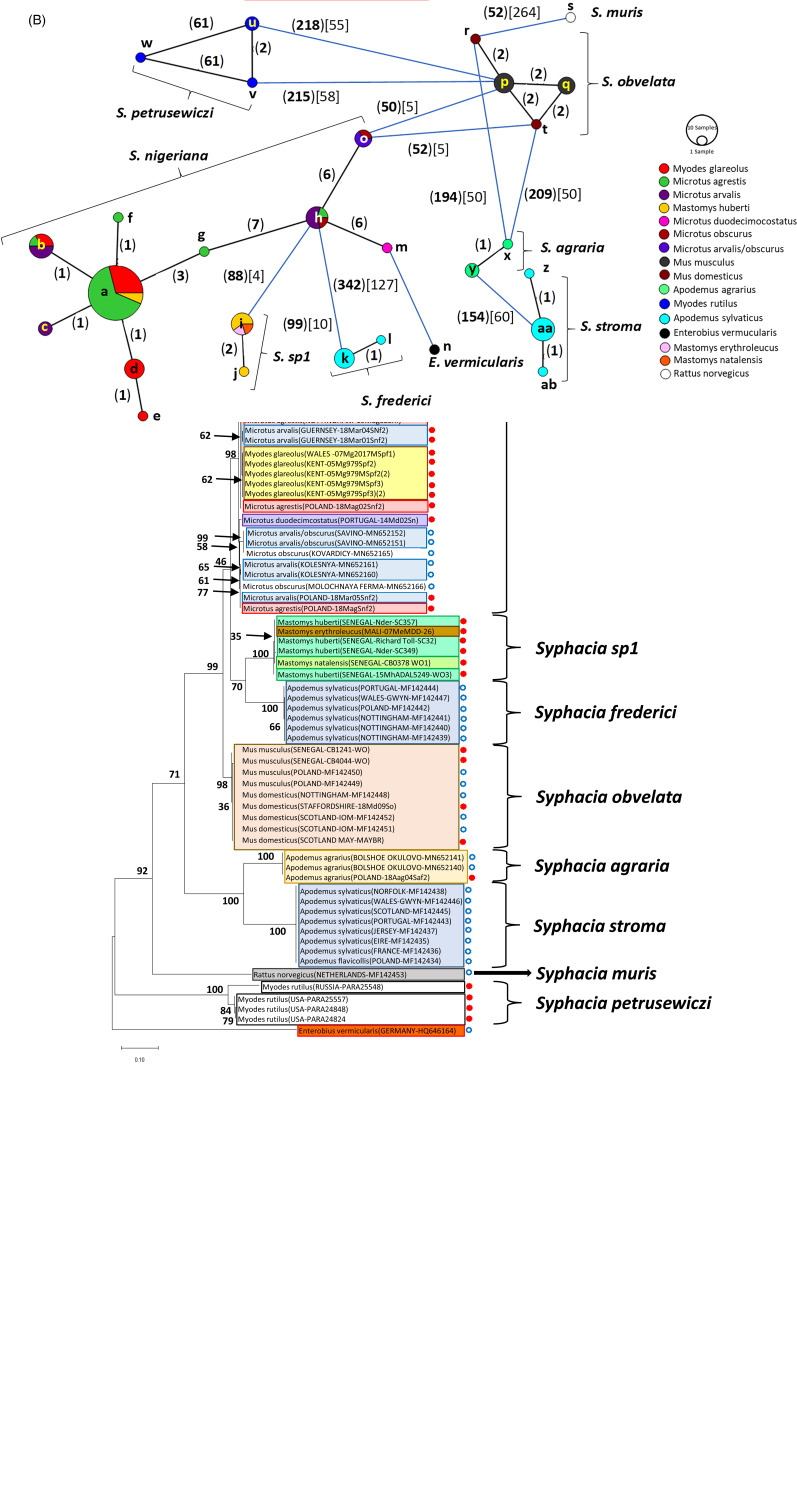
Molecular phylogenetic analysis of rDNA (ITS-1-5.8S-ITS2). (A) Molecular phylogenetic tree of *Syphacia* from murid and cricetid hosts following maximum likelihood (ML) analysis with 1000 bootstrap replicates. Scores at junctions represent bootstrap support for that junction. Scale bar is proportional to the genetic distance in substitutions per site. New sequences are marked by a red filled in circle and those taken from GenBank in blue open circles. (B) Minimum spanning network analysis. Bold numbers in round brackets on the lines show the number of single nucleotide polymorphisms between nodes and those in regular font and square brackets give the number of indels. Colours represent different rodent host species, and the newly genotyped isolates in each lettered node are listed in Table 1.

**Fig. 3.**
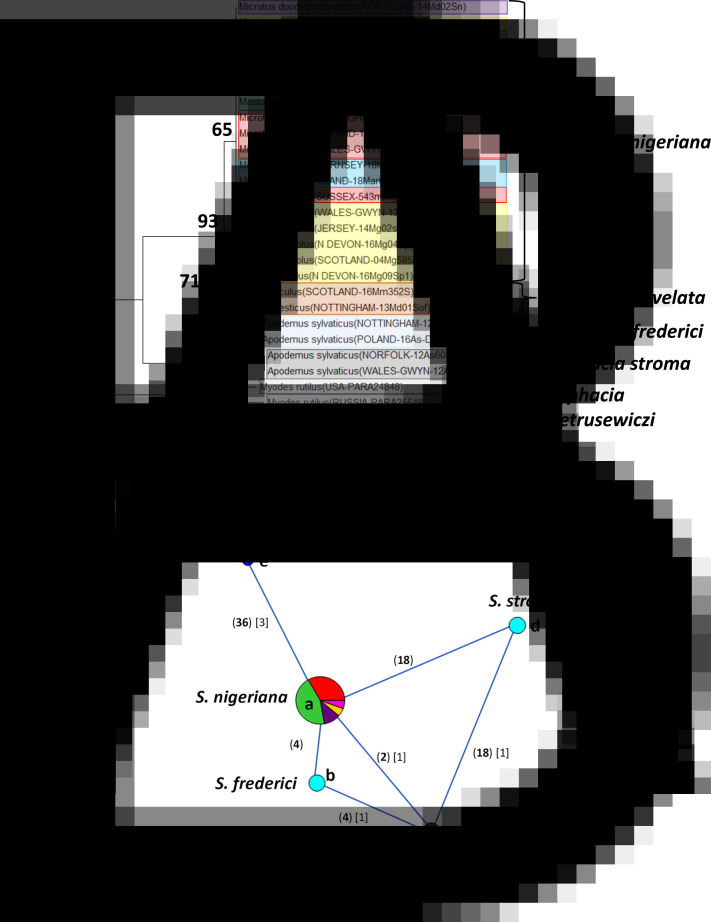
Molecular phylogenetic analysis of 18S rDNA (SSU). (A) Molecular phylogenetic tree of *Syphacia* from murid and cricetid hosts following maximum-likelihood (ML) analysis with 1000 bootstrap replicates. Scores at junctions represent bootstrap support for that junction. Scale bar is proportional to the genetic distance in substitutions per site. (B) Minimum spanning network analysis. Bold numbers in round brackets on the lines show the number of single nucleotide polymorphisms between nodes and those in regular font and square brackets give the number of indels. Colours represent different rodent host species (see legend to Fig. 2B for key), and the newly genotyped isolates in each lettered node are listed in Table 1.

**Fig. 4.**
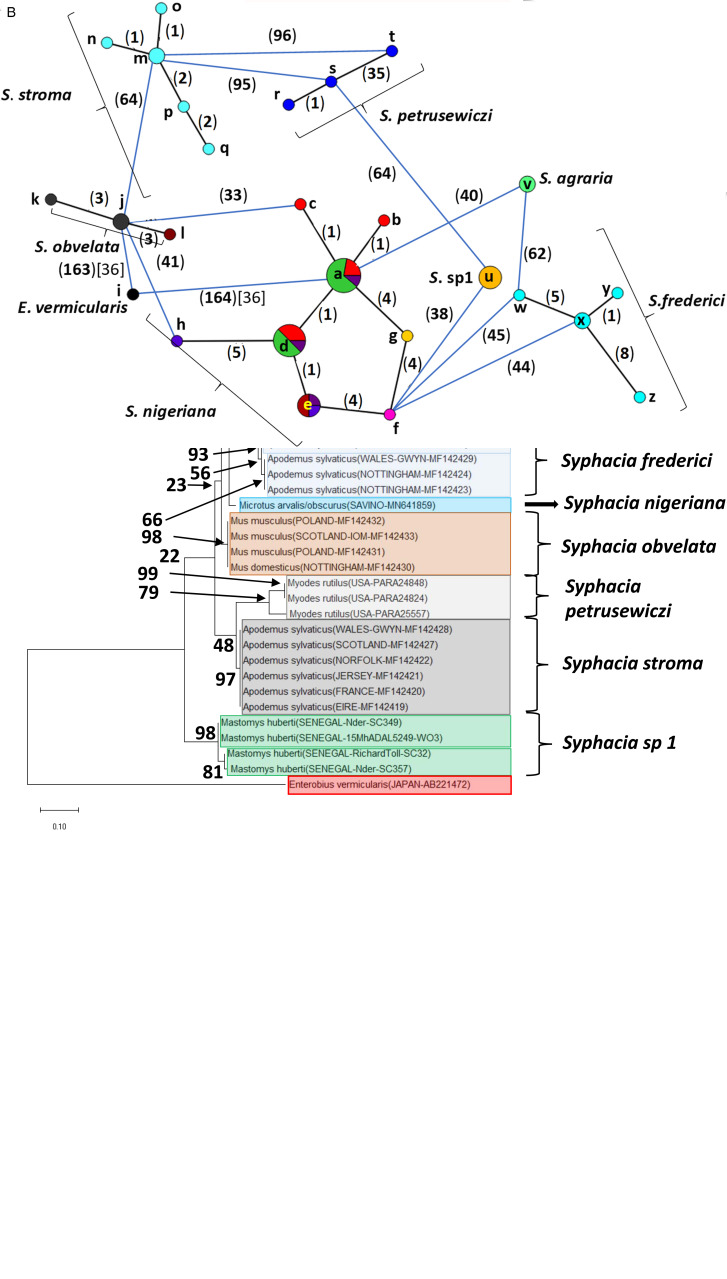
Molecular phylogenetic analysis of the *cox-1* gene. (A) Molecular phylogenetic tree of *Syphacia* from murid and cricetid hosts following maximum-likelihood (ML) analysis with 1000 bootstrap replicates. Scores at junctions represent bootstrap support for that junction. Scale bar is proportional to the genetic distance in substitutions per site. (B) Minimum spanning network analysis. Bold numbers in round brackets on the lines show the number of single nucleotide polymorphisms between nodes and those in regular font and square brackets give the number of indels. Colours represent different rodent host species (see legend to Fig. 2B for key), and the newly genotyped isolates in each lettered node are listed in Table 1.

### Molecular genetic comparison of isolates

DNA was isolated from individual worms using Extracta™ DNA prep kits (Quantabio) or DirectPCR lysis buffer (Viagen Biotech) according to the manufacturer's instructions. The *cox-1* gene was amplified using primers (Forward: 5′-TGGTCTGGTTTTGTTGGTAGTT-3′, Reverse: 5′-AACCACCCAACGTAAACATAAA-3′; Okamoto *et al*., 2007) spanning part of the coding sequence and yielding up to 684 bp amplicons, although some were truncated because of PCR amplification issues. The rDNA region consisting of ITS-1, 5.8S gene and ITS-2 (~700–750 bp) was amplified using the universal NC5 forward (5′-GTAGGTGAACCTGCGGAAGGATCATT-3′) and NC2 reverse primers (5′-TTAGTTTCTTTTCCTCCGCT-3′; Newton *et al*., 1998, Table 2). rDNA samples that failed to amplify were further analysed with nested forward (5′-GCTGTTTTCTTACATGCTATAAACG-3′) and reverse (5′-TATCATTACGTGGTTGACAGACA-3′) primers. The 18S rDNA (SSU) region was amplified using primers nSSU_F_03 (5′-GCTTGTCTCAAAGATTAAGCCATGC-3′) and nSSU_R_24 (5′-CCCCRRTCCAAGAATTTCACCTC3′; Blaxter *et al*., 1998 and http://www.nematodes.org/research/barcoding/sourhope/nemoprimers.shtml), resulting in a maximum amplicon length of 781 bp.

**Table 2.** Sequences from GenBank included in this study

Host	Nematode	GenBank reference	Country	Region	ITS^a^	*cox-1*^a^
***Apodemus* spp.**	***Syphacia* spp.**					
*A. agrarius*	*S. agraria*	MN641846	Russia^b^	Bolshoe-Okulovo	–	v
*A. agrarius*	*S. agraria*	MN641867	Russia	Bolshoe-Okulovo	–	v
*A. agrarius*	*S. agraria*	MN652140	Russia	Bolshoe Okulovo	y	–
*A. agrarius*	*S. agraria*	MN652141	Russia	Bolshoe Okulovo	y	–
*A. sylvaticus*	*S. stroma*	MF142419	Eire	Limerick, Foynes	–	m
*A. sylvaticus*	*S. stroma*	MF142420	France	Brittany	–	q
*A. sylvaticus*	*S. stroma*	MF142421	Jersey	Le Braye	–	p
*A. sylvaticus*	*S. stroma*	MF142422	England	Norfolk	–	n
*A. sylvaticus*	*S. frederici*	MF142423	England	Nottinghamshire	–	x
*A. sylvaticus*	*S. frederici*	MF142424	England	Nottinghamshire	–	y
*A. sylvaticus*	*S. frederici*	MF142425	England	Nottinghamshire	–	z
*A. sylvaticus*	*S. frederici*	MF142426	Portugal	Pancas	–	w
*A. sylvaticus*	*S. stroma*	MF142427	Scotland	Edinburgh	–	o
*A. sylvaticus*	*S. stroma*	MF142428	Wales	Gwynedd, Anglesey	–	m
*A. sylvaticus*	*S. frederici*	MF142429	Wales	Gwynedd, Anglesey	–	x
*A. flavicollis*	*S. stroma*	MF142434	Poland	Mazury	aa	–
*A. sylvaticus*	*S. stroma*	MF142435	Eire	Limerick, Foynes	aa	–
*A. sylvaticus*	*S. stroma*	MF142436	France	Brittany	aa	–
*A. sylvaticus*	*S. stroma*	MF142437	Jersey	Le Braye	aa	–
*A. sylvaticus*	*S. stroma*	MF142438	England	Norfolk	aa	–
*A. sylvaticus*	*S. frederici*	MF142439	England	Nottinghamshire	k	–
*A. sylvaticus*	*S. frederici*	MF142440	England	Nottinghamshire	k	–
*A. sylvaticus*	*S. frederici*	MF142441	England	Nottinghamshire	k	–
*A. sylvaticus*	*S. frederici*	MF142442	Poland	Mazury	k	–
*A. sylvaticus*	*S. stroma*	MF142443	Portugal	Pancas	ab	–
*A. sylvaticus*	*S. frederici*	MF142444	Portugal	Pancas	l	–
*A. sylvaticus*	*S. stroma*	MF142445	Scotland	z	–	
*A. sylvaticus*	*S. stroma*	MF142446	Wales	Gwyned, Angelsey	aa	–
*A. sylvaticus*	*S. frederici*	MF142447	Wales	Gwyned, Angelsey	k	–
***Homo sapiens***	***Enterobius* sp.**					
*Homo sapiens*	*E. vermicularis*	HQ646164	Germany	n	–	
*Homo sapiens*	*E. vermicularis*	AB221472	Japan	–	i	
***Microtus* spp.**						
*M. arvalis*	*Syphacia* sp.	MN641856	Russia^b^	Kolesnya	–	e
*M. arvalis/obscurus*	*Syphacia* sp.	MN641859	Russia	Savino	–	h
*M. obscurus*	*Syphacia* sp.	MN641860	Russia	Molochnaya Ferma	–	e
*M. obscurus*	*Syphacia* sp.	MN641862	Russia	Gremyachevo	–	e
*M. arvalis/obscurus*	*Syphacia* sp.	MN641866	Russia	Lesnikovo	–	e
*M. arvalis/obscurus*	*Syphacia* sp.	MN652151	Russia	Savino	o	–
*M. arvalis/obscurus*	*Syphacia* sp.	MN652152	Russia	Savino	o	–
*M. arvalis*	*Syphacia* sp.	MN652160	Russia	Kolesnya	h	–
*M. arvalis*	*Syphacia* sp.	MN652161	Russia	Kolesnya	h	–
*M. obscurus*	*Syphacia* sp.	MN652165	Russia	Kovardicy	o	–
*M. obscurus*	*Syphacia* sp.	MN652166	Russia	Molochnaya Ferma	h	–
***Mus* spp.**						
*M. domesticus*	*S. obvelata*	MF142430	England	Nottinghamshire	–	l
*M. musculus* (lab)^c^	*S. obvelata*	MF142431	Poland	Warsaw	–	j
*M. musculus* (lab)	*S. obvelata*	MF142432	Poland	Wrocław	–	j
*M. domesticus*	*S. obvelata*	MF142433	Scotland	Isle of May	–	k
*M. domesticus*	*S. obvelata*	MF142448	England	Nottinghamshire	t	–
*M. musculus* (lab)	*S. obvelata*	MF142449	Poland	Warsaw	p	–
*M. musculus* (lab)	*S. obvelata*	MF142450	Poland	Wrocław	p	–
*M. domesticus*	*S. obvelata*	MF142451	Scotland	Isle of May	q	–
*M. domesticus*	*S. obvelata*	MF142452	Scotland	Isle of May	q	–
***Rattus* spp.**						
*R. norvegicus*	*S.muris*	MF142453	Netherlands	Friesland	s	–

a Letters correspond to nodes in Figs 2B and 4B. Within each column isolates with the same letter have identical sequences.

b For further details of isolates from Russia recorded in GenBank, see Gorelysheva *et al*. (2021).

c (lab) These were BALB/c mice from the Universities of Warsaw and Wrocław

PCR reactions used Phusion Taq Polymerase according to manufacturer's instructions with 0.5 *μ*m of the forward and reverse primers, <250 ng of template DNA and nuclease-free water to a total volume of 25 *μ*L. Thermal cycling conditions for *cox-1* were: denaturation for 30 s at 98°C, then 35 cycles of 30 s at 98°C, 1 min at 52°C and 1 min 30 s at 72°C, with a final extension time of 5 min at 72°C before being held at 4°C. Thermal cycling conditions for the rDNA, rDNA-Nested and SSU regions were identical to *cox-1* with the exception of an annealing temperature of 60°C in both cases. Amplification in all PCR reactions was confirmed by visualization on a 1x SYBRSafe™ stained 1.5% agarose gel. PCR products were purified using AMPureXP beads according to the manufacturer's instructions and the final DNA concentration was estimated by Nanodrop before dilution with nuclease-free water to the required concentration for sequencing. Sequencing primers, identical to the amplification primers, were diluted to the required concentration with nuclease-free water and supplied to Source Bioscience or Eurofins, along with PCR products, for Sanger sequencing. Chromatograms were inspected visually for ambiguities and sequences trimmed to the 3′ end of the primers.

Sequence alignments were prepared using ClustalW within the MEGA X package (Kumar *et al*., 2018) followed by visual inspection. Phylogenetic analysis was performed in MEGA X using maximum likelihood (ML) with a partial deletion threshold set at 95% in MEGA (v10). Intraspecies (each separate parasite species based on phylogenetic analysis) and interspecies (all sequences) minimum spanning network (MSN) plots were produced in PopART (Leigh and Bryant, 2015) with an epsilon of zero (Bandelt *et al*., 1999). Intraspecies MSNs were linked together based on the interspecies MSN, but indel and single nucleotide polymorphism (SNP) numbers were added in manually given that sites with indels are ignored in MSN model calculations.

In addition to the novel sequences generated in this project, we exploited genetic records of rDNA and *cox-1* from *Syphacia* in GenBank. Some of these were from our own earlier work (Stewart *et al*., 2018), and those for *S. agraria* and isolates from *Microtus* spp. from Russia were from the work of Gorelysheva *et al*. (2021). Since the focus of the current study was on *Syphacia* spp. in *Microtus* spp., we used only representative sequences from *Apodemus* and *Mus* spp. but more information on how other isolates from these hosts fit into the phylogenetic trees can be found in Stewart *et al*. (2018). Because of the large number of identical sequences deposited by Gorelysheva *et al*. (2021) for isolates from Russian voles, we selected for the current analyses only representative sequences for the clades referred to in Gorelysheva *et al*. (2021), in order to show how our novel sequences related to those from Russian rodents.

Voucher sequences, including all sequences generated by this study and included in the current paper, have been deposited in GenBank (Table 1). Voucher numbers for accession to worms from *M. rutilus* held at the Museum Southwestern Biology, University of New Mexico, are as follows: Isolate from Russia, MSB:Para:25548; isolate from Alaska, USA, MSB:Para 25557; isolates from the Yukon, USA, MSB:Para:24824 and 24848.

### Microscopical analysis of isolates

All the specimens from hosts collected for this study and selected for morphological examination were cleared in lactophenol and viewed as temporary wet mounts using an Olympus BH-2 compound microscope. Light micrographs were taken using the same microscope. Measurements in micrometres were taken with the aid of an ocular micrometer and are given as the range where more than two measurements were taken. *En face* and transverse sections of the anterior body of representative specimens were prepared using a cataract scalpel and mounted in polyvinyl lactophenol for examination. All such specimens examined morphologically for this study, except those used for sectioning, were deposited in the South Australian Museum (SAMA) Adelaide, South Australia (Voucher numbers for specimens from Senegal are AHC 48827–48830, and all others AHC 48791–48826 and AHC 48831–48837).

## Results

### Molecular genetic comparison of worms

In total, we provide 118 new sequences [64 for rDNA (ITS-1-5.8S-ITS-2), 28 for *cox-1* and 26 for 18S rDNA (SSU)], which we have combined with 52 sequences from GenBank [29 for rDNA (ITS-1-5.8S-ITS-2) and 23 for *cox-1*].

### rDNA (ITS-1-5.8S-ITS-2) region

The ML phylogenetic tree for rDNA (ITS-1-5.8S-ITS-2), illustrated in Fig. 2A, shows that *Syphacia* isolates from rodents trapped throughout the British Isles had mostly identical genetic sequences, irrespective of whether the worms were isolated from field, bank or common voles (the latter only from Guernsey). A large proportion of the isolates from voles from the British Isles were identical, and these included isolates from the island of N. Uist in the Outer Hebrides, the most northerly location, to those from Devon in the south west of the British Isles, the island of Anglesey in Gwynedd, Wales in the west and Sussex in the southeast (Node **a** in Fig. 2B, and Table 1). This particular genotype was also identified in field voles from Poland and somewhat unexpectedly in two mice (*Mastomys huberti*) from Senegal in West Africa (Table 1). The latter was scrutinized very carefully to ensure that there was no possibility of any ambiguity in this finding.

Our data also show that there were some close variants of the node **a** sequence (Fig. 2B), including isolates from Kent (node **d)**, Nottingham (node **f**), Jersey, Derbyshire, Poland (node **b**) and Guernsey (node **c**), differing by just one SNP in each, but a different locus, and from Powys in Wales [differing by an additional SNP (node **e**, Fig. 2B)]. Not surprisingly, some *S. nigeriana* from more distant locations showed greater SNP variation when compared to the main *S. nigeriana* clade [e.g. some worms from Poland showed more SNPs (up to 3, node **g**), Russia (10-16 SNPs, nodes **o** and **h**) and *Microtus duodecimcostatus* from Portugal (16 SNPs, node **m**)]. However, given the length of the amplicon (860 bp), these were all relatively minor variations within what appears to be a single species of *S. nigeriana*, when compared, for example, to *S. muris* which differed from the main *S. nigeriana* clade (node **a**) by 93 indels and 240 SNPs.

*Syphacia* isolates from North American and Russian *M. rutilus* formed their own distinct and distant clade (node **u**, **v** and **w** in Fig. 2B), differing by 215 SNPs and 58 indels from that representing *S. obvelata* (node **p**). All the worms from *M. rutilus* had been identified previously as *S. petrusewiczi* (New Mexico Museum). Isolates from other specimens of *Mastomys* formed their own distinct clade (Fig. 2A) and branch (Fig. 2B, nodes **i** and **j**) and likely represent a new, as yet undescribed *Syphacia* species from West African *Mastomys* spp. (referred to hereafter as *Syphacia* sp1). We recovered these genotypes of *Syphacia* from three different species of *Mastomys*. As Fig. 2A and B shows other recognized species of *Syphaci*a (*S. stroma*, *S. frederici*, *S. obvelata*, *S. muris* and *S. agraria*) all formed their own distinct clades (Fig. 2A) and nodes (Fig. 2B) differing from node **a** isolates mostly by over 50 SNPs and in all cases also by indels. The only additional novel sequence in the current work was for *S. agraria* from a striped field mouse from Poland (Fig. 2B, node **x**), which proved to have a sequence that differed by just one SNP from that reported from the same host species from Russia (node **y**), and for an *S. obvelata* from the Senegal (Fig. 2B, node **p**) which had an identical sequence to isolates from laboratory mice from Poland but differed only by 2 SNPs from *S. obvelata* isolates from Scotland (node **q**) and by a different set of 2 SNPs from an isolate from Nottingham (node **t**).

### 18S rDNA (SSU)

Our analysis of the 18S rDNA region generated a phylogenetic tree that was largely congruent with that for the rDNA (ITS-1-5.8S-ITS-2) region (Fig. 3A). All the sequences we obtained from field, common (only on Guernsey) and bank voles, from locations in the British Isles were identical (Fig. 3B, node **a**). No variants were identified (Fig. 3A), pointing to this clade being a single species, i.e. *S. nigeriana*. Moreover, the 18S rDNA sequence from the isolate from *M. huberti* from the Senegal [that had an identical rDNA (ITS-1-5.8S-ITS-2) sequence to *S. nigeriana*] was clearly assigned to node **a** in Fig. 3B (and Table 2). The representative sequences from other recognized *Syphacia* spp. that we included in this analysis formed their own distinct clades with very high bootstrap values for junctions in the tree. Although sequence variation compared to *S. nigeriana* (node **a**) ranged from just 2 SNPs and 1 indel to *S. obvelata*, and then 18 SNPs and 1 indel to *S. stroma* (Fig. 3B), the greatest comparative sequence variation was for *S. petrusewiczi* from *M. rutilus* from North America (36 SNPs) and Russia (45 SNPs) and 3 indels in both cases compared to *S. nigeriana*.

### cox-1

Analysis of the *cox-1* locus produced a more complex picture (Fig. 4A and B). Again, it is clearly apparent that sequences from worms from many of the field, common and bank voles from the British Isles were identical or differed by just 1 SNP (nodes **a** and **d** in Fig. 4B). These included worms from *M. agrestis* and *M. arvalis* from Poland. In this analysis, the isolate from *M. huberti* (SENEGAL-10MeKB6461-W03), that had an identical rDNA (ITS-1-5.8S-ITS2*)* sequence to the *S. nigeriana* in node **a** (Fig. 2B), differed in its *cox-1* sequence from the main *S. nigeriana* clade (node **g** in Fig. 4B) by 4 SNPs. The *cox-1* sequence of the isolate from *M. duodecimcostatus* from Portugal (node **f**) differed from those in node **a** by 8 SNPs and those in node **d** by 5 SNPs. As expected, all the other recognized *Syphacia* spp. showed greater discrepancies in SNPs, each species forming a distinct clade in Fig. 4A, although some of the bootstrap values were low. Consistent with their status as a new species, the four isolates from *M. huberti* that had differed from *S. nigeriana* in rDNA sequences also had their own distinct sequence for the *cox-1* locus (node **u** in Fig. 4B). As in the rDNA, *S. petrusewiczi* formed a distinct distant clade of its own (Fig. 4A), although the isolate from Alaska (USA-PARA25557, node **t**) differed by 35 SNPs from the Yukon isolate (USA-PARA24824; Fig. 4B, node **s**) and there was a single SNP distinguishing the two Yukon isolates (USA-PARA24824 *vs* USA-PARA24848).

### Morphological comparison of specimens

All the worms examined from British and European localities, regardless of host species and locality, were identified as *S. nigeriana* on the basis of their morphology and measurements of key morphological features (Table 3). No consistent differences were found in the morphometrics of males or females between samples, as shown in Table 3. Characters which showed variability between individual samples of females included egg size and shape but such differences could not be related to a particular host species. For example, the longest eggs were found in an individual of *M. arvalis* from Guernsey, up to 128 *μ*m and an individual of *M. agrestis* from the West Midlands of England, up to 132 *μ*m long. The shortest eggs measured were from an individual of *M. agrestis* from Nottingham, 99 *μ*m. All other egg lengths varied within these limits, showing no particular size pattern. Similarly, female tail length varied between 390 and 900 *μ*m long, shorter tail lengths being found in immature females regardless of the origin of the sample. The same variability was seen in measurements of the distance between the anterior end and the vulva. By contrast, the morphometrics of the male samples showed less variability across locality and host species (Table 3).

**Table 3.** Isolates of *Syphacia* species examined microscopically

Reference	Host	No. examined	Length (mm)	Vulva – anterior (*μ*m)	Tail (*μ*m)	Egg dimensions (*μ*m)
**Female worms from *Mastomys* spp.**						
SENEGAL-10MeKB6341-W02	*M. huberti*	1	4.6	657	650	105.6−108.9 × 29.7−33
SENEGAL-10MeKB6461-W02	*M. huberti*	1	4.4	737	800	118.8 × 33
SENEGAL-15MhADAL5249-W02	*M. huberti*	10	3.1–3.8	302	550–800	118.8−122.1 × 33−36.3
SENEGAL-04MnCBO 378-W02	*M. natalensis*	2	3.1–4.8	503–530	900	118.8 × 33−36.3
**Female worms from *Microtus* spp.**						
DORSET-467mMagSnf1	*M. agrestis*	6	4.1–4.6	483–737	460–750	105.6−112.2 × 23.1−29.7
GLOUCESTERSHIRE-449mMagSnf2	*M. agrestis*	2	3.4, 3.6		550	108.9−112.2 × 23.1−33
GLOUCESTERSHIRE-780mMagSnf1	*M. agrestis*	2	5.1, 5.7	737, 663	690, 700	108.9−115.5 × 26.4−29.7
LANCASHIRE-1067M5nf	*M. agrestis*	4	3.2–4.0	1445	500–640	Immature
NORTHUMBERLAND-16MaV229Snfb	*M. agrestis*	10	2.5–3.8	363–700	456–703	102−125 × 33−36
NOTTINGHAM-18Mag 065nf1	*M. agrestis*	2	3.3. 3.7	429	600, 700	99 × 23.1
OXFORDSHIRE-783 MagSnf1	*M. agrestis*	2	5.4	518, 705	500, 400	105.6-112.2 × 26.4−33
POLAND-13Mag07b5n	*M. agrestis*	3	3.1–4.2	556	610	Immature
POLAND-18Mag02Snf1	*M. agrestis*	10	4.9–6.0	650–790	670–737	105.6−118.8 × 29.7−33
POLAND-18MagSnf1	*M. agrestis*	10	4.6–5.1	436–657	703–906	105.6−108.9 × 33−39.6
SCOTLAND-14Ma015n	*M. agrestis*	11	3.4–5.4	637–950	637–800	119–125 × 26−33
STAFFORDSHIRE-894MagSnf1	*M. agrestis*	1	3.9	536	700	108.9 × 33
SUSSEX-543mMagSnf1	*M. agrestis*	4	3.6–4.6	300–415	410–710	105.6 × 33
WALES-GWYN-17Mag05Sn2	*M. agrestis*	10	0.98–1.5	363–555	436–623	Immature
W MIDLANDS-447mMag Snf1	*M. agrestis*	2	5.2	436	420, 700	125.4−132 × 33
WILTSHIRE-995Msn/f1	*M. agrestis*	15	3.7–5.3	536	`670–726	118−125 × 26−33
GUERNSEY-18Mar015n/f1	*M. arvalis*	11	4.0–5.3	690–905	549–704	109−128 × 33−36
GUERNSEY-18Mar04Sn/f1	*M. arvalis*	5	4.5–5.8	670–1038	665–737	125.4 × 36.3
POLAND-13Mar55Sn	*M. arvalis*	3	4.5, 5.0	540, 550	800,900	106−116 × 25−33
POLAND-16Mr04Sn	*M. arvalis*	3	4.9	680	510	109,106 × 33
POLAND-18Mar05Snf	*M. arvalis*	2	5	820	703	102.3−105.6 × 29.7−33
POLAND-Mar32Snf	*M. arvalis*	10	2.5–3.2	363–570	390–469	Immature
PORTUGAL-14Md02Sn	*M. decimcostatus*	3	3.4–3.7	590–670	460–610	119−129 × 30−36
**Female worms from *Myodes* spp.**						
JERSEY-14Mg02sp	*M. glareolus*	6	4.2–4.6	550–1445	670–770	118−125 × 30−46
N.DEVON-16Mg09Sp2	*M. glareolus*	3	3–3.3	402–482	603–690	122−125 × 30−33
KENT-05Mg979MSpf1	*M. glareolus*	5	4.0–4.3	570, 636	590–600	
**Male worms**				**Spicule**		**Gubernaculum**
DORSET-467mMagSnm	*M. agrestis*	11	1.02–1.6	51–81.6	100–170	25.5–51
GLOUCESTERSHIRE-780mMagSnm	*M. agrestis*	1	-	69.7		30.6
POLAND-18Mag02Snm	*M. agrestis*	1	1.15	76.5	90	37.4
WILTSHIRE-995Msnm	*M. agrestis*	5	1.4–1.6	75.9–85.8	155–181	33–36.3
GUERNSEY-18Mar015 nm	*M. arvalis*	10	1.1–2.3	66–92.4	148–178	33–42.9
POLAND-Mar32Snm	*M. arvalis*	3	1.3–1.5	69.7–85	110–180	34–36
KENT-05Mg979MSpm	*M. glareolus*	1	1.3	83	-	35.7

**Fig. 5.**
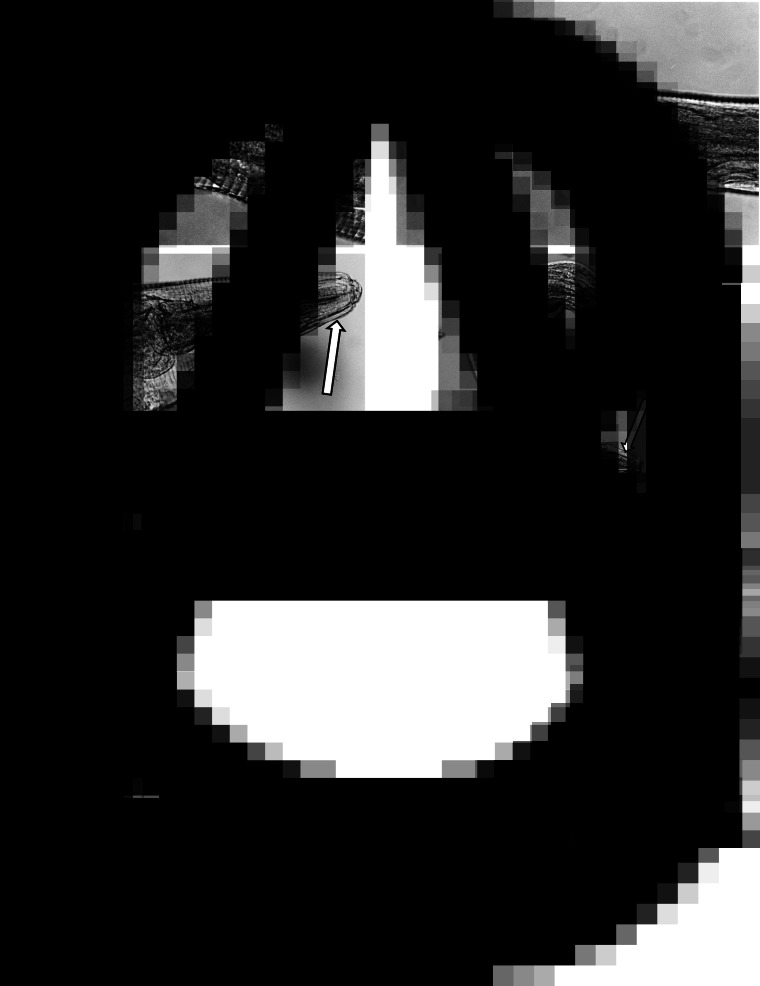
(A) Lateral view of the anterior end of female *Syphacia nigeriana* from *Microtus agrestis* (NORTHUMBERLAND 16MaV229Snfb), showing the cephalic vesicle (arrow). (B) Lateral view of the anterior end of a female *S. nigeriana* from *M. agrestis* (Wales GWYNLLANB 17Mag05Sn2), showing the cephalic vesicle (large arrow) and non-protruding vulva (small arrow). (C) Lateral view of the anterior end of a female *S. nigeriana* from *M. agrestis* (POLAND – 18MagSnf1), showing the cephalic vesicle (large arrow) and protruding vulva (small arrow). (D) Lateral view of male *S. nigeriana* (DORSET-467mMagSnm), showing the cephalic vesicle (large arrow) and mamelons (thin arrow). Scale bars A and C = 75 *μ*m; B = 150 *μ*m; D = 120 *μ*m.

Nor could any consistent morphological differences be found between samples. All of the specimens had the typical key characters of *S. nigeriana*, including the cephalic structures, absence of cervical alae and presence of narrow lateral alae, that are used to differentiate both males and females of the species. The cephalic vesicle was a prominent feature of most but not all specimens although it should be noted that this character may be adversely affected by the fixation protocol used (Fig. 5A and B). The only character that showed some morphological variation was the shape of the vulva (Fig. 5B and C). Some Polish specimens from *M. arvalis* had an ornamented vulva, some Polish specimens from *M. agrestis* had a flap covering the vulva, and some specimens from *M. glareolus* from North Devon had a protruding vulva.

None of the specimens had ornamented cervical alae or lacked lateral alae, key characters for *S. petrusewiczi.* Males of *S. nigeriana* from this study had spicules up to 92 *μ*m long, compared with up to 56 *μ*m, a tail up to 180 *μ*m long compared with up to 66 *μ*m long and the first mamelon up to 550 *μ*m from the anterior end compared with up to 300 *μ*m for *S. petrusewiczi* (Tenora and Mészáros, 1975).

**Fig. 6.**
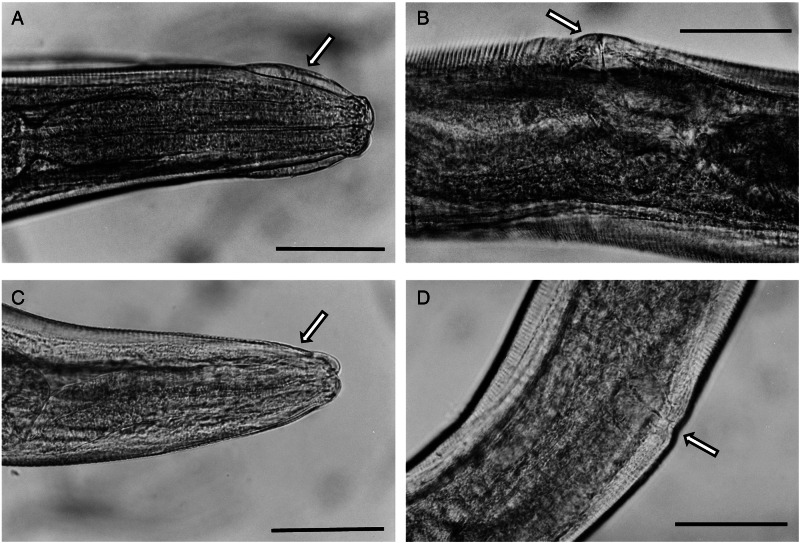
Photomicrograhs of *Syphacia nigeriana* and *Syphacia* sp.1 from *Mastomys huberti* from Senegal. (A and B) *S. nigeriana* (10MeKB6341-W02); arrow in A indicates the cephalic vesicle and in B the dome-shaped vulva. (C and D) *Syphacia* sp.1 (15MhADAL5249-W02); arrows in C show the lack of a cephalic vesicle and in D the non-ornamented vulva. Scale bars are 100 *μ*m.

Single specimens were available from each of two *M. huberti* SENEGAL-10MeKB6341-WO2 and SENEGAL-10MeKB6461-WO2 and no significant differences in the morphometrics between these samples and *S. nigeriana* could be found (Fig. 6A and B), so from morphometrics and microscopical examination of visible features, both were congruent with *S. nigeriana*. However, morphometric examination of the females from a single individual of *Mastomys natalensis* (2 specimens; SENEGAL-04Mn CB0378-W02) and three individuals of *M. huberti* (12 specimens; SENEGAL-15MhADAL5249-W02; Table 3), showed that whilst having morphometrics consistent with *S. nigeriana*, these worms differed in not having a cephalic vesicle, and alae that could be interpreted as either no cervical alae, lateral alae beginning anteriorly or no break or differentiation between lateral and cervical alae. The vulva of these females was unobtrusive and not ornamented. Some specimens had the vulva slightly protruding and some resembled the vulva of specimens of *S. nigeriana* from the Welsh collection of worms.

Comparisons between an *en face* preparations of a specimen from an individual of *M. huberti* (SENEGAL-15MhADAL5249-WO2) from Senegal, and *M. agrestis* from Northumberland (NORTHUMBERLAND-16MaV229Snfb) and from Wiltshire (WILTSHIRE-995Msn/f1) indicated that this *M. huberti* was harbouring a different species. All the specimens had an oval cephalic plate, extended laterally with submedian papillae and a mouth with three lips, and could therefore be placed in Group V of Quentin (1971). This group includes species of *Syphacia* from rodents from central and north Africa and the Holarctic. The cephalic plate of the specimen from *M. huberti* was shaped differently from that typical for *S. nigeriana*. The distance between the amphids was smaller (35 as compared to 50, 55 μm for the 2 specimens from *M. agrestis*) and the lips were also proportionally smaller.

## Discussion

When we began this project, informed by the robust and often repeated statements of earlier workers that in Europe *S. petrusewiczi* is the dominant *Syphacia* infecting rodents of the genus *Myodes* (e.g. *M. glareolus*) and *S. nigeriana* those of the genus *Microtus* (Tenora and Mészáros, 1975; Mészáros, 1978), we had expected to see on our phylogenetic trees at least two clearly separated genetic clades for each of the genetic loci we have been sequencing, one associated with worms from bank voles and the other for worms from field and common voles of the genus *Microtus*. Moreover, given the well-established co-evolution of *Syphacia* spp. with their hosts (Hugot, 1988), and the enormous geographical barriers to gene flow between Nigeria in West Africa and Europe, we were also sceptical about the likelihood that a species of *Syphacia* originally described from five species of murid rodents from Nigeria (two species in the subfamily Gerbillinae and three in the subfamily Murinae) could be the same or even very closely related to a common species infecting voles (Cricetidae, Arvicolinae) in Europe. Our data show, however, that on both counts we were wrong.

Our phylogenetic analysis shows clearly that at the genetic level, based on two genetic regions [rDNA (ITS-1-5.8S-ITS2) and 18S rDNA (SSU)] and one locus (*cox-1*) *Syphacia* worms recovered from bank voles and from field and common voles from the British Isles, all formed one genetic clade and not two distinct clades as we had expected. Irrespective of the host, or the site from which the hosts had been sampled throughout the British Isles and parts of Europe, and implementing Occam's razor, we conclude that the worms whose genes we sequenced were sufficiently close genetically to be considered just one species and not two. Moreover, all measurements of the specimens of *S. nigeriana* from both Europe and the British Isles were congruent with those previously reported in descriptions of *S. nigeriana*, both of the Type (Baylis, 1928) and other material from African hosts, and material from European hosts (see e.g. Tenora and Mészáros, 1975). Their cephalic structures and key morphological characters were all congruent with the analyses of Quentin (1971) and Hugot (1988) for *S. nigeriana.*

Unexpectedly, the gene sequences of two worms, each from a different individual of *M. huberti*, a species that is closely related to one of the hosts from which Baylis (1928) had originally described *S. nigeriana* in Nigeria (*M. erythroleucus*), proved to be virtually identical or very similar to those from our *Myodes*/*Microtus* isolates from the British Isles. This finding lends support to Quentin's (1971) conclusion that indeed *S. nigeriana*, as described by Baylis (1928), is also a parasite of European *Microtus* spp., and based on our results and those of earlier morphology-based studies, also that of *Myodes* spp.

Our results raise several relevant issues. Firstly, earlier authors have reported what they considered to be morphological features that differ between *S. petrusewiczi* and *S. nigeriana*. As emphasized by Wiger *et al*. (1978), *S. petrusewiczi* has prominent cervical alae, whereas *S. nigeriana* does not. This was also underlined by Hugot (1988) whose tables indicate that *S. petrusewiczi* is typified by prominent and ornamented cervical alae. Careful microscopical examination of representative worms from our collection did not reveal evidence of prominent cervical alae on any of the worms from British *Myodes* or *Microtus*. Furthermore, *S. petrusewiczi* does not have lateral alae whereas *S. nigeriana* has narrow lateral alae and males of *S. petrusewiczi* have a short tail compared to the males of *S. nigeriana* which have a longer tapering tail (Tenora and Mészáros, 1975). Since males, however, are usually not found at dissection, the opportunity to distinguish throughout between the two species based on male characters was not possible. Several of the features that distinguish the two species are based on scanning electron microscopical (SEM) studies (a row of denticles on each of the three main teeth of *S. nigeriana* and their lack in *S. petrusewiczi* (Wiger *et al*., 1978); differences in the topographical surface of the eggs, especially in the shape and outline of the operculum (Barus *et al*., 1979)]. It will be apparent that features that can only be identified through SEM do not lend themselves well to quantitative studies of parasite burdens, and it is therefore not surprising that identification of worms by previous authors relied largely on the host rather than on specific morphological features that differ between worm species.

Secondly, although we failed to find any worms from bank voles from the British Isles and Europe that differed markedly from those we had sequenced from *Microtus* spp., we were able to include in our genetic analysis worms that had been clearly identified as *S. petrusewiczi rauschi*, derived from eastern Russian and North American *M. rutilus*. In accordance with Okamoto *et al*. (2009), these had genetic sequences that were markedly different from those we had obtained from worms from European voles, and formed their own distinct genetic clade, with only minor SNP variation between isolates from Russia and from North America. Isolates of *S. petrusewiczi* from Russia have also been found recently to form their own distinct clade based on sequences of the rDNA (ITS-1-5.8S-ITS-2) and the large subunit 28S regions (Gorelysheva *et al*., 2020), although to date the sequences are not available in GenBank. Our isolates from Russia and North America had been carefully examined by one of us (JMK) and designated as *S. petrusewiczi rauschi* and deposited as such in the Museum Southwestern Biology, University of New Mexico. It is interesting that this *S. petrusewiczi rauschi* clade was quite distant to that of our worms from European voles with a deep split in the phylogenetic tree of each of the three genes in our study. This is exactly consistent with the cladistic tree published by Hugot (1988) in which *S. petrusewiczi* and *S. petrusewiczi rauschi* were illustrated as sister species on a branch deeply separated from that on which *S. nigeriana* and *S. obvelata* were placed. It is also consistent with Gorelysheva *et al*. (2020) and Okamoto *et al*. (2009), the latter concluding that since *S. petrusewiczi* formed a deep split in the phylogenetic tree, it diverged much earlier than the other rodent pinworms examined in their study. Hugot (1988) erected three subgenera within the genus based on the morphology he had characterized. As a result, *S. nigeriana* was placed in the subgenus *Syphacia* and *S. petrusewiczi* in the subgenus *Seuratoxyuris*, thus reinforcing the morphological separation of the two species, and early divergence of *S. petrusewiczi* from other rodent species (Okamoto *et al*., 2009). The close relationship between *S. nigeriana* and *S. obvelata*, that was apparent in Hugot'as (1988) cladistic tree, was also clearly evident in our results. In Hugot's (1988) cladistic study, specimens of *S. nigeriana* were not derived from European *Microtus* spp., but from *Hylomyscus stella* (Thomas, 1911), the Stella wood mouse from the Republic of Central Africa. So it is possible that the worms he examined may have been the original species that Baylis (1928) described as *S. nigeriana*, or a close relative.

Thirdly, nematodes of the genus *Syphacia* are among the most widespread helminth species infecting wild rodents (Roman, 1951), but the different species are also difficult to distinguish, based on morphological characters as traditionally applied. This is partly because much of the taxonomy is based on male worms which are infrequently encountered, or even extremely rare in some species (see e.g. Mészáros, 1978, who found only females of *S. petrusewiczi*), and are very delicate and small, but nevertheless are character richer than females. Differences between female worms, which dominate parasite burdens, are mostly minor and difficult to recognize. The difficulties inherent in distinguishing between species of *Syphacia* are reflected in the literature in studies where worms were first ascribed to *S. petrusewiczi* (Wiger *et al*., 1976), followed by recantation of their identity, and subsequently re-identification as *S. nigeriana* (e.g. Tenora *et al*., 1977). Where parasite burdens are heavy (*Syphacia* worm burdens in mice can exceed several thousand/host), examination of each individual is an onerous task, and we suspect that many authors may have relied simply on the host species as the key determinant of the *Syphacia* they may have been infected with. Moreover, in many reports, it is just not clear what specific characters were used to distinguish between *S. petrusewiczi* and *S. nigeriana* (e.g. Tenora *et al*., 1991) and how reliable these may have been. Although we do not know of any studies quantifying variation in morphological characters between individuals of a specific *Syphacia* sp., it is well established that morphological characters vary in size, shape and even presence or absence within species of animals, and no less so among nematodes [see e.g. Le Jambre (1977) and Hunt *et al*. (2008), and their studies on variation in the morphology of the vulvar flaps of *Haemonchus contortus*].

Fourth, we were surprised that gene sequences from worms from *M. huberti* fell clearly within our clade from European *Microtus*. Whilst this is consistent with the idea that this West African rodent shares the same parasite as European *Microtus* and *Myodes*, we were not able to extend this to the other species of rodents in Baylis' study. As our project progressed we were able to sample worms from a range of other West African rodents and our results will be reported elsewhere, as they raise yet more issues.

Fifthly, despite extensive efforts during this work to detect some evidence that *S. petrusewiczi* exists in British rodents, we were unable to find this species. The genetic sequences that we obtained from Russian *M. rutilus* and the recent study by Gorelysheva *et al*. (2020) indicate that it does exist in Eurasian *Myodes* spp., but we were unable to detect its genetic signature in any of the worms we sampled from bank voles from the British Isles. It is instructive that the worms from bank voles on the island of Jersey (which does not have any *Microtus* spp.) also proved to have the genetic signature of *S. nigeriana*. In contrast on the island of Guernsey there are no wild bank voles, the only species of vole is *M. arvalis*, and the *Syphacia* from these hosts differed from those on Jersey by just a single SNP. It may be that we were just unlucky in failing to find *S. petrusewiczi* in British bank voles, and that an even more extensive trapping and sampling campaign may have found the species, but given our widespread sampling, this does not sit easily with Tenora and Mészáros (1975), who emphasized that *S. petrusewiczi* is a dominant parasite of *M. glareolus*. They also concluded that *S. nigeriana* is a parasite of both *Microtus* spp. and more rarely *M. glareolus*, which is consistent with our study. In further studies, Tenora *et al*. (1977, 1979*b*) reported *S. nigeriana* from Norwegian bank voles but found only very low abundance with this species. These authors emphasized again that *S. nigeriana* is essentially a parasite of *Microtus* spp., but may infect bank voles in particular cases.

Finally, our results lead us to conclude that *S. nigeriana* is indeed a species which is less host-specific than, for example, *S. stroma* (only found in *Apodemu*s spp.) or *S. obvelata* (only found in *Mus* spp.). *Syphacia nigeriana* has been recorded from a range of *Microtus* spp., not just in Europe but also in North America (Quentin, 1971), as well as in other rodent species in Africa (Baylis, 1928; Quentin, 1971). Based on our genetic analysis, currently restricted to British and West African rodents, it appears to exist among these hosts as a species complex with several genetic variants that differ in just a few SNPs from each other but not sufficiently to merit different species status. Its host range in Africa may be even wider, but that remains to be confirmed. On this basis, *S. nigeriana* must be considered a generalist that has switched hosts on more than one occasion, spreading to new rodent hosts within the cricetid and murid families, and has become the dominant species in European voles. We found no evidence that *S. petrusewiczi* exists in bank vole populations living in the British Isles but we can now confirm that the only report of *S. nigeriana* in field voles from England (Turner *et al*., 2014) was correct, since our sequences from worms from field voles from Northumberland (including from Kielder Forest) were identical to those in clades that we concluded were *S. nigeriana*.
